# Differential effects of ganglioside lipids on the conformation and aggregation of islet amyloid polypeptide

**DOI:** 10.1002/pro.5119

**Published:** 2024-07-16

**Authors:** Samuel D. McCalpin, Lina Mechakra, Magdalena I. Ivanova, Ayyalusamy Ramamoorthy

**Affiliations:** ^1^ Biophysics Program University of Michigan Ann Arbor Michigan USA; ^2^ Department of Chemistry University of Michigan Ann Arbor Michigan USA; ^3^ Department of Neurology University of Michigan Ann Arbor Michigan USA; ^4^ Michigan Neuroscience Institute University of Michigan Ann Arbor Michigan USA; ^5^ Biomedical Engineering, Macromolecular Science and Engineering University of Michigan Ann Arbor Michigan USA; ^6^ National High Magnetic Field Laboratory, Department of Chemical and Biomedical Engineering, Institute of Molecular Biophysics, Neuroscience Florida State University Tallahassee Florida USA

**Keywords:** amylin, amyloid, ganglioside, IAPP, lipid

## Abstract

Despite causing over 1 million deaths annually, Type 2 Diabetes (T2D) currently has no curative treatments. Aggregation of the islet amyloid polypeptide (hIAPP) into amyloid plaques plays an important role in the pathophysiology of T2D and thus presents a target for therapeutic intervention. The mechanism by which hIAPP aggregates contribute to the development of T2D is unclear, but it is proposed to involve disruption of cellular membranes. However, nearly all research on hIAPP‐lipid interactions has focused on anionic phospholipids, which are primarily present in the cytosolic face of plasma membranes. We seek here to characterize the effects of three gangliosides, the dominant anionic lipids in the outer leaflet of the plasma membrane, on the aggregation, structure, and toxicity of hIAPP. Our results show a dual behavior that depends on the molar ratio between the gangliosides and hIAPP. For each ganglioside, a low‐lipid:peptide ratio enhances hIAPP aggregation and alters the morphology of hIAPP fibrils, while a high ratio eliminates aggregation and stabilizes an *α*‐helix‐rich hIAPP conformation. A more negative lipid charge more efficiently promotes aggregation, and a larger lipid headgroup improves inhibition of aggregation. hIAPP also alters the phase transitions of the lipids, favoring spherical micelles over larger tubular micelles. We discuss our results in the context of the available lipid surface area for hIAPP binding and speculate on a role for gangliosides in facilitating toxic hIAPP aggregation.

## 1 INTRODUCTION

Type 2 Diabetes (T2D) is a top 10 cause of global mortality and accounts for over 1 million deaths annually (Ong et al., 2023). While treatments exist to manage symptoms of T2D, such as insulin resistance and reduced insulin secretion, no cure currently exists (Nauck et al., 2021). The prevalence of T2D is on the rise, particularly in developed nations, which emphasizes the pressing need for treatments that target its root cause (Ong et al., 2023). While the pathology of T2D is multifactorial, protein aggregation in the islets of the pancreas plays an important role in reducing insulin‐secretion capacity by destroying insulin‐secreting *β*‐cells (Marzban et al., 2003). T2D belongs to a class of conditions called amyloidosis, in which amyloid plaques composed of fibrillar, *β*‐sheet‐rich protein aggregates form in affected tissues (Milardi et al., 2021). Islet amyloid primarily comprises aggregates of the islet amyloid polypeptide (IAPP), a 37‐residue peptide hormone that is expressed and secreted in response to elevated blood glucose, alongside insulin (Betsholtz, Svensson, et al., 1989; Milardi et al., 2021; Mosselman et al., 1989; Nishi et al., 1989; Sanke et al., 1988). A wealth of evidence links IAPP aggregation to the development of T2D. For example, animals that have an aggregating variant of IAPP will spontaneously develop diabetes, while animals with IAPP nonaggregating variants do not develop diabetes (Betsholtz, Christmansson, et al., 1989; Hoenig, 2012; Howard, 1978; Milardi et al., 2021). Furthermore, diabetes can be induced in these animals by transgenic modification to express human IAPP (hIAPP) (Janson et al., 1996; Matveyenko & Butler, 2006). Aggregating variants of IAPP are also toxic to cultured cells while nonaggregating variants are not (Palato et al., 2019). However, the toxicity does not derive from the mature amyloid fibrils (Abedini et al., 2016; Janson et al., 1999; Kapurniotu, 2001). Rather, intermediate aggregates, or “oligomers,” appear to directly cause toxicity to cells (Haataja et al., 2008).

Consensus has not been reached on a mechanism of IAPP cytotoxicity, but leading theories implicate interactions between IAPP and lipid membranes. IAPP binds to and permeabilizes lipid bilayers containing anionic lipids, so it has been proposed that IAPP oligomers might disrupt plasma membranes (Raleigh et al., 2017; Zhang, St Clair, et al., 2017). Some studies suggest that IAPP can penetrate cell membranes and form *β*‐barrel pores that act as ion channels, leading to a disruption of membrane integrity, imbalanced cellular homeostasis, and cell death (Sciacca et al., 2021; Sepehri et al., 2021). Alternative hypotheses posit that IAPP oligomers disrupt lipid membranes via a detergent‐like mechanism, siphoning lipids from the membrane surface, or by direct interaction with protein receptors in the cell membrane, causing a signaling cascade that results in apoptosis (Abedini et al., 2018; Tempra et al., 2022). Regardless, proximity to or direct interaction with cellular membrane lipids appears to mediate IAPP toxicity in cells.

However, most of the research in this area has utilized model membranes containing anionic phospholipids. While phospholipids and cholesterol are the dominant classes of lipids in cell membranes, anionic phospholipids primarily localize in the cytosol‐facing membrane leaflets (Di Paolo & De Camilli, 2006; Dodge & Phillips, 1967; Ingólfsson et al., 2014; van Meer et al., 2008; Verkleij et al., 1973; Virtanen et al., 1998; Zwaal et al., 1975). Thus, these previous studies may not provide sufficient insight into the interaction between IAPP and the outer leaflet of the plasma membrane. This is potentially an issue because intracellular IAPP is stored in secretory granules and thus unlikely to encounter cytosol‐facing membrane surfaces (Hickey et al., 2009). Instead, IAPP might interact with anionic lipids in the extracellular face of the plasma membrane, such as gangliosides. Gangliosides are glycosphingolipids composed of a ceramide tail and an oligosaccharide headgroup that contains at least one sialic acid moiety (Kolter, 2012). They are also the major anionic lipid component of the outer leaflet of plasma membranes, comprising 15 mol% of the outer leaflet lipids in red blood cells (Doktorova et al., 2023; Feizi, 1985; Ingólfsson et al., 2014; Op den Kamp, 1979). When cholesterol is abundant in the plasma membrane, gangliosides form clusters known as rafts. Ganglioside‐containing lipid rafts have a high negative‐charge density to attract positively charged membrane‐binding proteins, like IAPP.

Gangliosides have been implicated in amyloid formation associated with Alzheimer's Disease (AD) and Parkinson's Disease (PD), but relatively little is known about their role in islet amyloidosis (Ledeen & Wu, 2018; Matsuzaki, 2014). It is notable that there is considerable uncertainty in the ganglioside content of pancreatic cells relevant to T2D. Whole human pancreas extracts mostly contain the gangliosides GM3, GD3, and GD1a (Dotta et al., 1989). But human pancreatic islets, which contain the insulin‐ and IAPP‐secreting *β*‐cells, are depleted in GM3, GD3, and GD1a compared to the whole pancreas and enriched in an unidentified GM2‐comigrating ganglioside (Dotta et al., 1989). While rat islet and whole pancreas ganglioside compositions varied greatly between the two studies, the islets possessed greater total ganglioside content relative to whole rat pancreas, suggesting that gangliosides are abundant in pancreatic islets (Dotta et al., 1993; Saito & Sugiyama, 2000). One study from Wakabayashi and Matsuzaki (2009) supports the involvement of gangliosides in mediating toxicity associated with aggregated IAPP. They found that disrupting ganglioside rafts in the cell membrane reduced amyloid formation and toxicity by hIAPP and that hIAPP did not aggregate or cause toxicity when incubated with cells that do not produce gangliosides (Wakabayashi & Matsuzaki, 2009). MD simulations similarly observed that monomeric IAPP bound on or near GM3 lipid rafts in a phospholipid membrane and that the GM3 clusters facilitated a helix‐to‐*β*‐sheet structural conversion of membrane‐bound IAPP (Christensen & Schiøtt, 2019).

**FIGURE 1:**
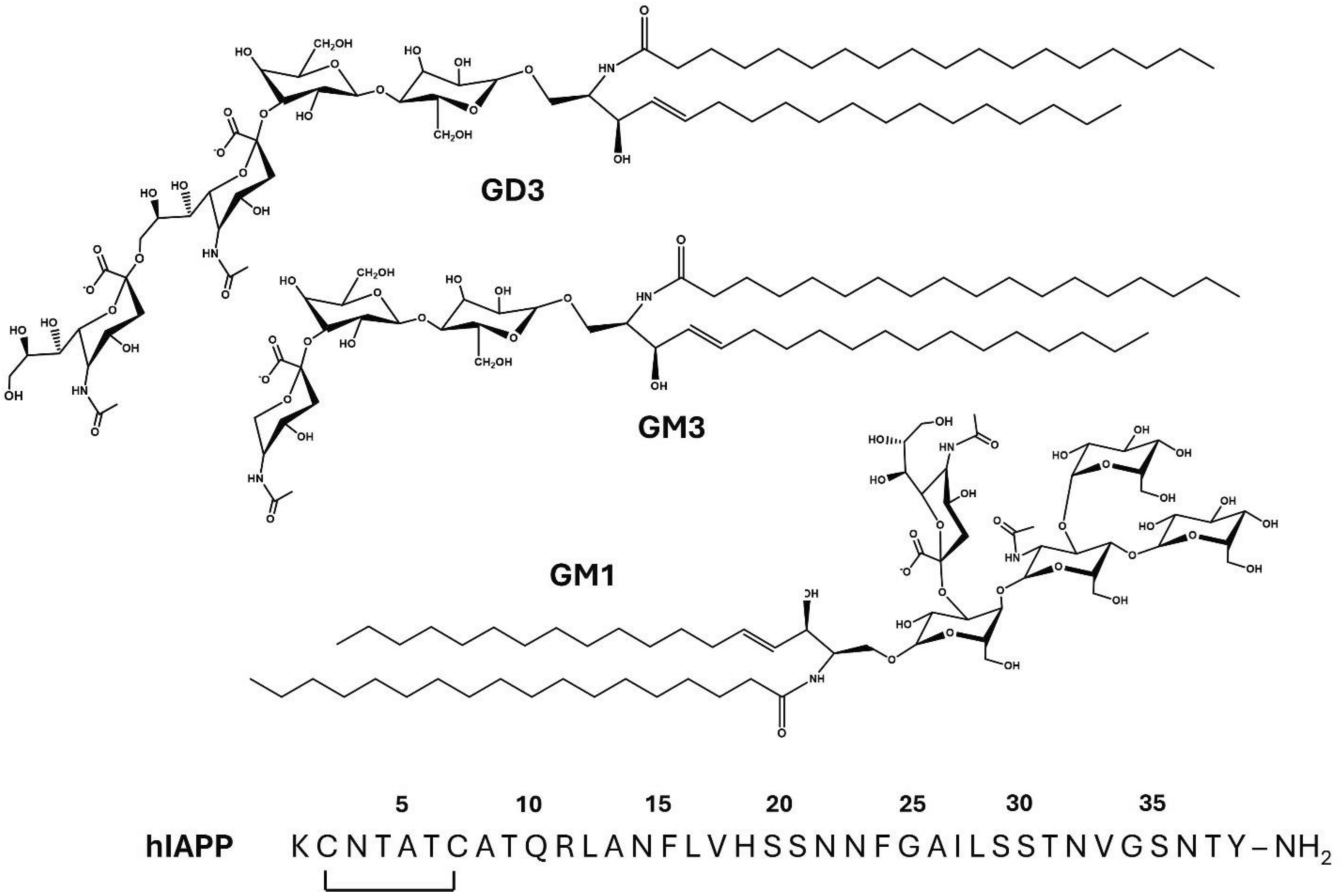
Molecular structures of ganglioside lipids and amino acid sequences of human islet amyloid polypeptide (hIAPP). The molecular structures for the noted gangliosides are shown, and the amino acid sequence of hIAPP is displayed with the intramolecular disulfide bond and amidated C‐terminus noted.

Though there is evidence to implicate gangliosides in islet amyloidosis, to our knowledge, there has been no experimental work to define the molecular details of an interaction between gangliosides and hIAPP. As such, we performed a biophysical characterization of hIAPP aggregation, structure, and morphology in the presence of ganglioside‐containing membranes. For our studies, we chose to work with three ganglioside lipids—GM1, GM3, and GD3 (Figure 1). GM3 and GD3 were selected because they are physiologically relevant to the pancreatic environment, and GM1 would allow a comparison to previous research with amyloid‐*β* (A*β*) and *α*‐Synuclein (*α*S), the amyloid peptides associated with AD and PD, respectively (Baba et al., 1998; Ledeen & Wu, 2018; Matsuzaki, 2014). We investigated the effects of each ganglioside on the aggregation behavior of hIAPP with kinetic fluorescence assays, CD spectroscopy, TEM, and cell viability assays. Our results demonstrate a dual, concentration‐dependent effect on hIAPP aggregation kinetics, conformation, and toxicity. We then discuss our findings in the context of mechanisms by which gangliosides could mediate IAPP toxicity.

## 2 METHODS AND MATERIALS

### 2.1 Materials

C‐amidated hIAPP (purity ≥95%) was purchased from Anaspec (Fremont, CA, USA). The gangliosides GM1 (ovine brain extract, sodium salt, catalog #860065), GM3 (bovine brain extract, ammonium salt, catalog #860058), and GD3 (bovine brain extract, ammonium salt, catalog #860060) were obtained from Avanti Polar Lipids (Alabaster, AL, USA). All other chemicals were purchased from Sigma Aldrich (St. Louis, MO, USA), except for thioflavin T (ThT), which was purchased from Cayman Chemicals (Ann Arbor, MI, USA).

### 2.2 Peptide and lipid sample preparation

Prior to use, hIAPP was dissolved in hexafluoro‐isopropanol (HFIP) to a concentration of 1 mM and allowed to incubate at room temperature for 1 h. hIAPP was aliquoted from this stock and lyophilized. Sample peptide concentrations were calculated based on these aliquots. Lipid stock solutions were prepared at 2.5 mg/mL in a 1:1 mixture of methanol and chloroform. Lyophilized hIAPP and lipid stocks were stored at −20°C until use. From the stock solutions, lipids were aliquoted, dried to a film under a nitrogen stream, and further dried under a vacuum overnight. These films were resuspended in aqueous buffer for 1 h and used immediately.

### 2.3 Thioflavin T fluorescence assay

All samples for the ThT fluorescence assay were prepared on ice, containing a sodium phosphate buffer (10 mM sodium phosphate, 100 mM NaCl, pH 7.4) with 20 μM ThT and the lipid concentrations noted in the figures. Immediately prior to beginning each measurement, lyophilized hIAPP was dissolved in the same phosphate buffer and added to samples for a final peptide concentration of 5, 10, or 20 μM. Samples were added to a black‐walled, 384‐well plate with a clear, flat bottom (Greiner catalog #07000892) in quadruplicate (30 μL per well). Fluorescence was measured without shaking in a FLUOstar Omega microplate reader (BMG Labtech Inc.) by exciting at 440 nm and measuring fluorescence at 490 nm every 8 min with gain set at 90%. The temperature inside the microplate reader was maintained at 25°C for the duration of the experiment. Fluorescence data is shown as the average of the four replicates per sample, with error bars representing one standard deviation. All ThT assays were independently repeated at least once to ensure reproducibility. Amylofit was used to calculate half‐times for each trial, and these were averaged and reported with one standard deviation error (Meisl et al., 2016).

### 2.4 Transmission electron microscopy

Following ThT fluorescence assays, hIAPP samples were collected for visualization of aggregates by TEM. Control samples containing only lipid (15 or 150 μM) were also prepared freshly as described above. Negatively stained specimens for TEM were prepared by applying 5 μL of sample to hydrophilic 400‐mesh carbon‐coated Formvar support films mounted on copper grids (Ted Pella, Inc., cat# 01702‐F). The samples were allowed to adhere for 4 min, washed twice with ddH2O, and stained for 60–90 s with 5 μL of 1% uranyl acetate (Ted Pella, Inc.). All samples were imaged at an accelerating voltage of 60 kV in a JEM 1400 Plus (JOEL). Images were collected from at least three grid regions at magnifications of 10,000x–60,000x, and representative micrographs were reported here.

### 2.5 Circular dichroism

Circular dichroism (CD) samples were prepared by mixing 50 μM hIAPP with 0, 75, or 750 μM ganglioside lipid in sodium phosphate buffer (10 mM sodium phosphate, 100 mM NaF, pH 7.4). Spectra were measured as an average of 10 accumulations with a Jasco CD spectrophotometer every hour for 24 h. The sample cell was maintained at 25°C for the duration of the experiments. Experimental parameters were 100 nm/min scanning speed, 1 nm bandwidth, 0.5 nm data pitch, 1 s data integration, and a 200 mdeg CD scale. Reference spectra were measured using samples without IAPP and automatically subtracted from the sample spectra. BestSel was used to estimate secondary structure contents (Micsonai et al., 2022). For samples with 50 μM hIAPP and 750 μM lipid, secondary structure estimates are reported as the average from all timepoints, with one standard deviation error.

### 2.6 Cell toxicity assay

Toxicity to rat pancreatic *β*‐cells was assessed with the MTT cell viability assay. RIN‐5F cells (ATC CRL‐2058, batch 61465080) were grown in RPMI‐1640 medium with GlutaMAX (diluted from 100x solution Fisher cat#35‐050‐061), 10% FBS, and Penicillin/Streptomycin at 37°C and 5% CO_2_. Cells were passaged a minimum of three splitting cycles after revival from frozen stocks prior to toxicity experiments and discarded after 25 passages. The MTT assay was performed with the Promega CellTiter 96 cell proliferation assay kit (Promega G4000). In a transparent 96‐well plate, 40,000 cells were added to wells in 90 μL of cell growth medium and allowed to adhere for 24 h. Samples were then added from 10x stocks, with five replicates per sample, to reach a final hIAPP concentration of 10 μM and final lipid concentrations of 10 or 100 μM. The sample plates were incubated for 48 h at 37°C and under 5% CO_2_. Per the manufacturer protocol, 15 μL of MTT dye solution was added to each sample, followed by 4 h of incubation. Then, 100 μL of stop solution was added to each well. Cell proliferation was assessed by measuring the difference between *A*_570_ and *A*_700_ for each well, averaging the replicates for each sample, subtracting the absorbance difference of a sample with 1% SDS, and normalizing relative to a buffer control. This experiment was performed independently three times, and cell viability was reported as an average of 15 sample replicates. A one‐way ANOVA test was used for statistical analysis (Jiang et al., 2022).

## 3 RESULTS

### 3.1 Ganglioside lipids exerted a dual effect on hIAPP aggregation

**FIGURE 2:**
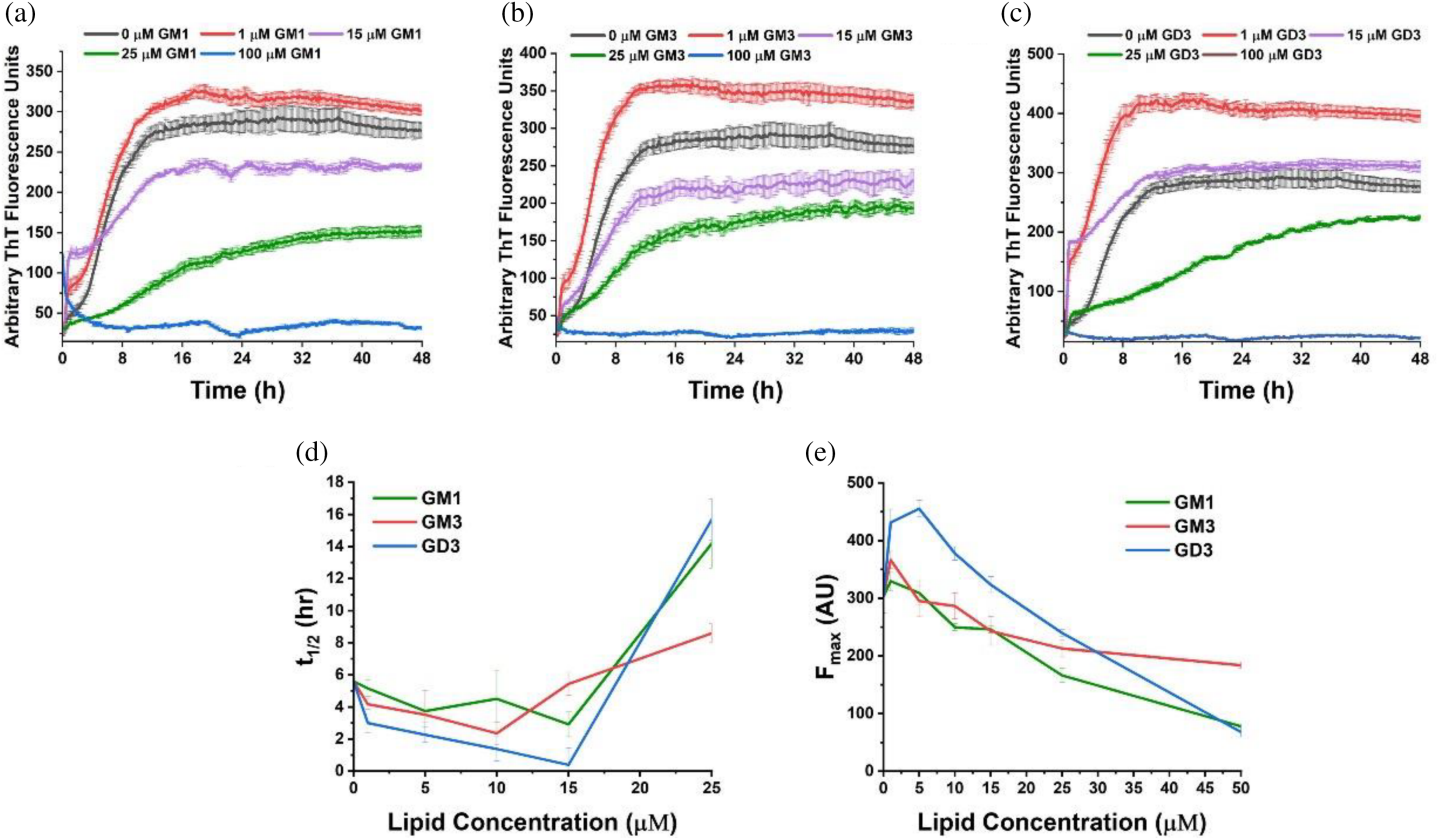
Aggregation kinetics of human islet amyloid polypeptide (hIAPP) with gangliosides monitored by thioflavin T (ThT) fluorescence. Samples contained 10 μM hIAPP, 20 μM ThT, 10 mM sodium phosphate, 100 mM NaCl, pH 7.4, and the noted concentrations of (a) GM1, (b) GM3, and (c) GD3. (d) Half‐times and (e) maximum fluorescence intensities were calculated for each condition and plotted versus lipid concentration.

To investigate the effects of ganglioside lipids on hIAPP aggregation, we performed ThT fluorescence assays with 10 μM hIAPP and a concentration series of GM1, GM3, and GD3. The ThT fluorescence data revealed two distinct behaviors of gangliosides on hIAPP aggregation (Figure 2). At low concentrations, gangliosides reduced the time to half‐maximal fluorescence (*t*_1/2_) and increased the maximum fluorescence intensity (*F*_max_). The greatest reduction in *t*_1/2_ was observed with a concentration of 15 μM for GM1 and GD3 and 10 μM for GM3. *F*_max_ was most increased with 1 μM lipid for GM1 and GM3 and 5 μM lipid for GD3. GD3 decreased *t*_1/2_ and increased *F*_max_ to a greater extent than either GM1 or GM3. At higher lipid concentrations, all three gangliosides increased *t*_1/2_ and reduced *F*_max_ to the baseline level. Both GM1 and GD3 eliminated the increase in ThT fluorescence with 50 μM lipid, while 100 μM GM3 was required to do the same.

**FIGURE 3:**
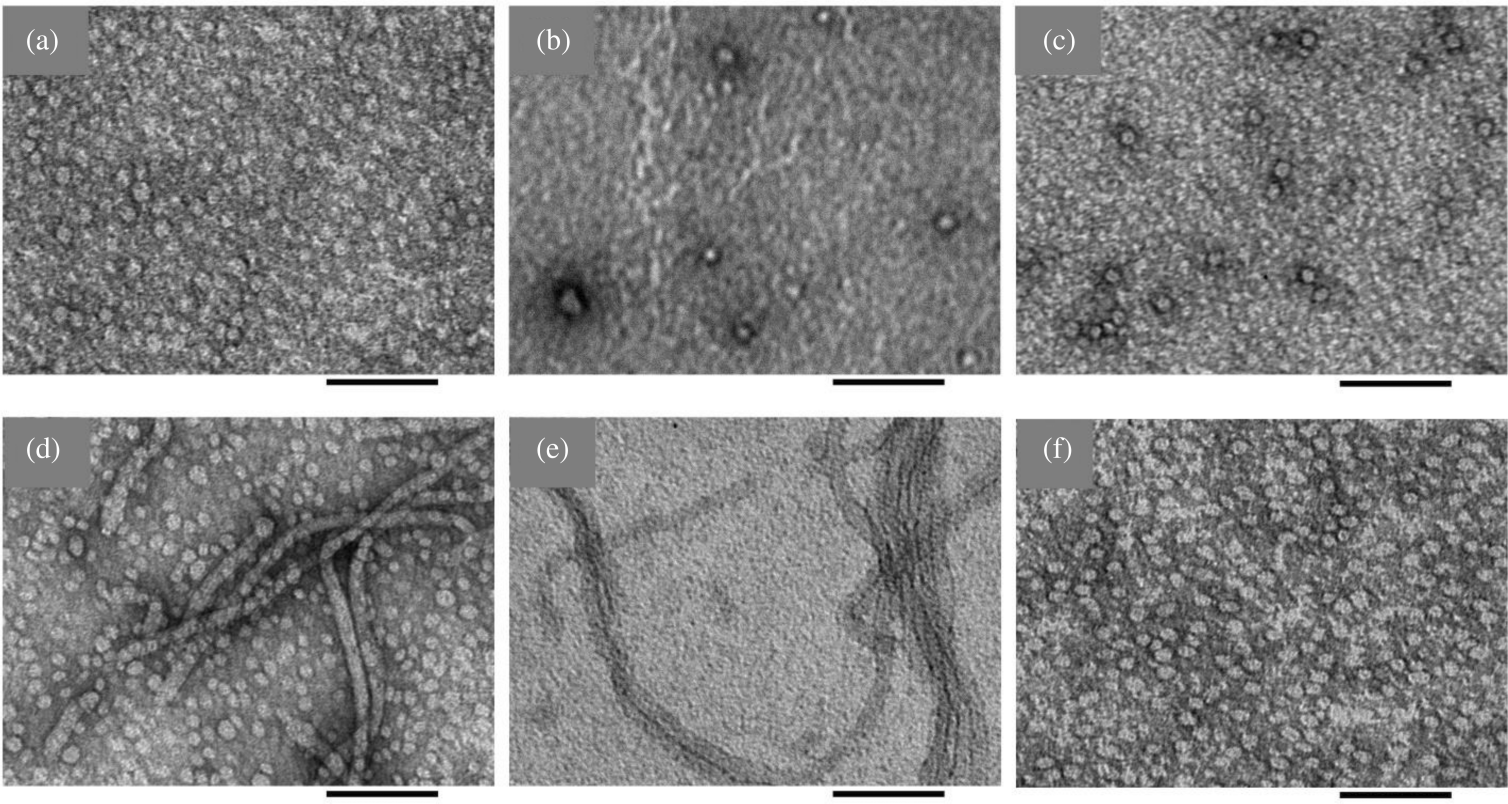
TEM micrographs of gangliosides. Samples were prepared with (a) 15 μM GM1, (b) 15 μM GM3, (c) 15 μM GD3, (d) 150 μM GM1, (e) 150 μM GM3, or (f) 150 μM GD3 in 10 mM sodium phosphate, 100 mM NaCl, pH 7.4. Scale bars = 100 nm.

**FIGURE 4:**
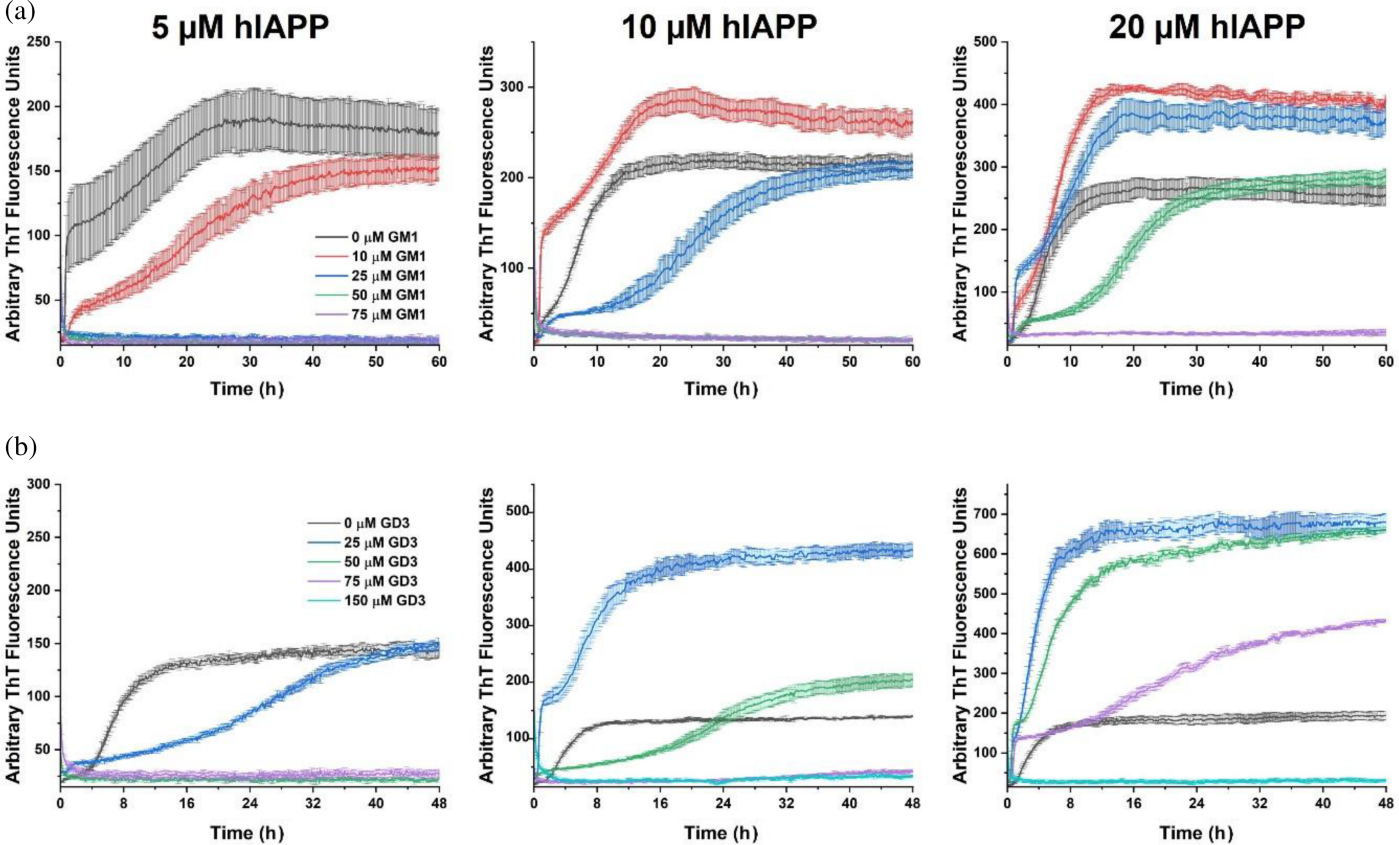
Human islet amyloid polypeptide (hIAPP) aggregation kinetics are dependent on the molar ratio between ganglioside and hIAPP. Thioflavin T fluorescence assays were performed with the noted concentrations of hIAPP and (a) GM1 or (b) GD3 in buffer containing 10 mM sodium phosphate, 100 mM NaCl, and pH 7.4. Data for GM3 is in Figure S1.

Previous work has measured a GM1 phase transition at a concentration in the range 10–100 μM that has been assigned to the critical micelle concentration (CMC) (Chakravorty et al., 2022; Oshima et al., 1993; Rauvala, 1979; Saha et al., 2023; Yohe & Rosenberg, 1972). However, these measurements relied on pyrene fluorescence or triiodide formation assays that report a change in the dielectric constant or hydrophobicity of the medium rather than a direct observation of micellar species and that can thus misrepresent the true CMC (Serravalle et al., 2024). Several reports using more direct measurements of particle sizes, such as sedimentation or light scattering, determined a sub‐micromolar CMC of GM1 (Corti et al., 1980, 1982; Formisano et al., 1979). We sought to characterize the lipid species present at different concentrations to clarify this discrepancy and the effect of the ganglioside phase on hIAPP aggregation. TEM micrographs (Figure 3) showed that spherical or discoidal micelles were present with 15 μM of each ganglioside. Increasing the concentrations of GM1 and GM3 to 150 μM resulted in the formation of worm‐like or tubular micelles, which potentially explains the lipid phase transition that was measured by pyrene fluorescence and misattributed to the CMC. At this concentration, GM1 formed a mixture of tubular and spherical micelles, while GM3 was entirely tubular. In contrast, the more negatively charged GD3 underwent no phase change between 15 and 150 μM, remaining entirely as spherical micelles, so it seemed unlikely that the catalytic and inhibitory effects of the gangliosides on hIAPP aggregation arose from distinct ganglioside species. Accordingly, further ThT experiments with different concentrations of hIAPP (Figure 4) demonstrated that the impact of gangliosides on hIAPP aggregation depended primarily on the lipid:peptide molar ratio rather than the lipid phase. The lipid:peptide molar ratio required to completely inhibit aggregation was greatest for GM3 and least for GM1, but the molar ratio for each lipid varied across replicates, between 4x and 10x lipid.

**FIGURE 5:**
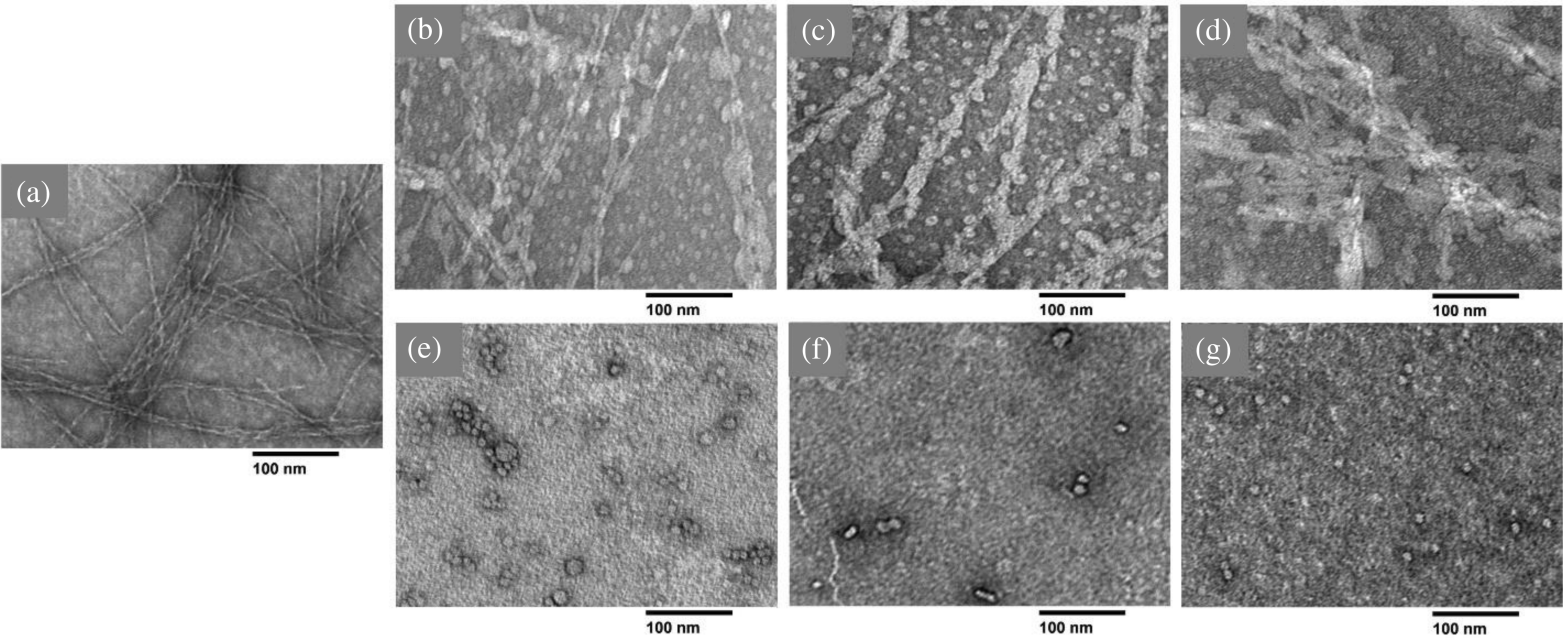
TEM micrographs of human islet amyloid polypeptide (hIAPP) incubated with gangliosides. Samples containing 10 μM hIAPP and (a) no lipids, (b) 15 μM GM1, (c) 15 μM GM3, (d) 15 μM GD3, (e) 150 μM GM1, (f) 150 μM GM3, or (g) 150 μM GD3 were monitored by Thioflavin T fluorescence for 96 h to confirm complete aggregation and stained with 1% uranyl acetate for TEM imaging.

To corroborate the effects of gangliosides on hIAPP aggregation observed by ThT fluorescence, we collected TEM micrographs of hIAPP samples after ThT fluorescence reached plateau (Figure 5). hIAPP alone formed homogeneous amyloid fibrils. The addition of 1.5x molar excess ganglioside induced the formation of thinner, straighter fibrils with additional amorphous density wrapped around the fibrils. These wrapped fibrils were morphologically similar with each lipid. Greater heterogeneity was observed among the lipid‐induced aggregates, with short, ribbon‐like assemblies of fibrils, and fuzzy amorphous aggregates also present (Figures 5, Figure S2). Increasing the ganglioside concentrations to a 15x molar excess relative to hIAPP eliminated fibril formation. For each of these samples, only round micelles were observed, despite GM1 and GM3 alone forming tubules at the same concentration.

### 3.2 Gangliosides induced conformational changes in hIAPP

**FIGURE 6:**
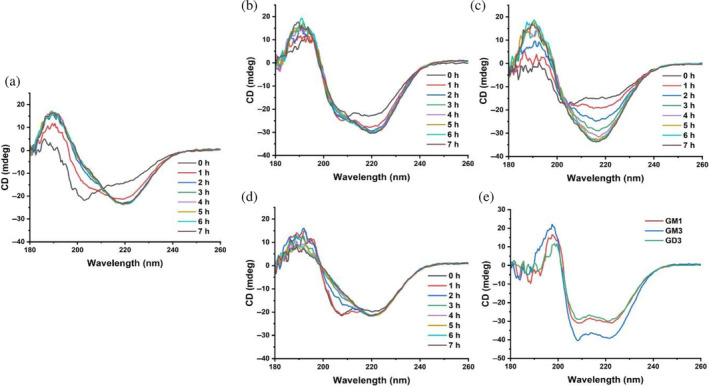
Time‐dependent circular dichroism (CD) spectra of human islet amyloid polypeptide (hIAPP) incubated with gangliosides. hIAPP (50 μM) was incubated with (a) no lipids, (b) 75 μM GM1, (c) 75 μM GM3, (d) 75 μM GD3, or (e) 750 μM of each ganglioside in a sodium phosphate buffer (10 mM sodium phosphate, 100 mM NaF, pH 7.4). Spectra were measured every hour for at least 20 h and are presented for (a–d) the first 8 h or (e) the first hour.

To investigate conformational changes of hIAPP in the presence of gangliosides, we followed the time course of hIAPP aggregation in fibril‐seeding (1.5x molar excess ganglioside) and fibril‐inhibiting (15x molar excess ganglioside) conditions with CD spectroscopy (Figure 6). The CD spectrum of hIAPP alone initially displayed a negative minimum at 201 nm, which shifted to 219 nm within a few hours, consistent with a transition from random coil to *β*‐sheet. In contrast, the addition of a 1.5x molar excess of any of the gangliosides resulted in double minima at 208 and 221 nm, indicative of *α*‐helix. Over time, these two minima transformed to a single minimum at 219 nm, indicating an *α*‐helix to *β*‐sheet structural conversion. A higher concentration of gangliosides (15x molar excess) produced a similar starting CD spectrum that was stable for at least 24 h (Figures 6E, Figure S3). We then deconvoluted the CD spectra of hIAPP with 15x lipid to estimate the secondary structure content of stable lipid‐bound hIAPP (Tables S1 and S2). The estimates indicated that, regardless of the ganglioside, the micelle‐bound hIAPP mostly contained *α*‐helix and random coil secondary structures. The CD deconvolutions also predicted smaller amounts of *β*‐sheet and turn structures that depended on the ganglioside identity. However, *β*‐sheet and turn content varied substantially based on the range of the CD data used in secondary structure estimations.

### 3.3 Cytotoxicity of ganglioside‐induced hIAPP aggregates

**FIGURE 7:**
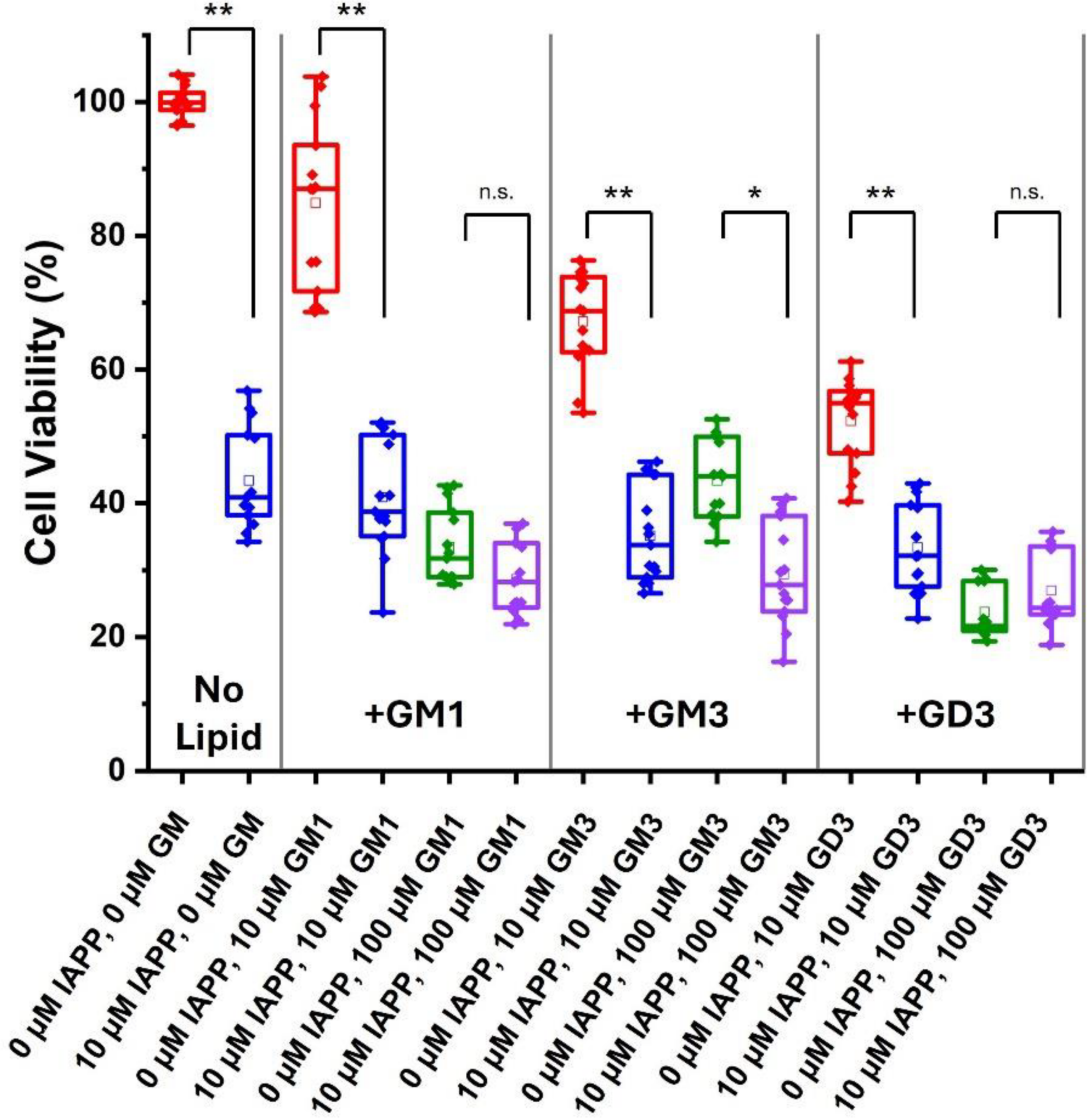
Viability of rat insulinoma cells incubated with human islet amyloid polypeptide (hIAPP) and gangliosides. Cell viability was measured by the MTT cell viability assay for RIN‐5F cells incubated with the noted samples for 48 h. The box and whisker plots show the sample median (solid line), mean (square), and scatter plot of raw data. A one‐way ANOVA test was used for statistical analyses. Pairwise differences between samples with (blue) and without (red) 10 μM hIAPP are denoted as *p* >0.05 (n.s.), 0.01< p <0.05 (*), or *p* <0.01 (**). All treatments except for 0 μM hIAPP, 10 μM GM1 (box 3) caused significantly reduced viability compared to the buffer control (box 1).

We incubated hIAPP and ganglioside lipids with RIN‐5F rat insulinoma cells and performed MTT cell viability assays to investigate the connection between ganglioside‐induced conformational changes of hIAPP and cytotoxicity (Figure 7). hIAPP alone was toxic after 48 h incubation, reducing cell viability by 57% compared to the buffer control. All the ganglioside treatments in the absence of hIAPP also decreased cell viability, except for 10 μM GM1, which caused a statistically insignificant reduction of cell viability. At a concentration of 100 μM, GD3 and GM1 were more toxic on their own than GM3, and the ganglioside toxicity was dose‐dependent, increasing with concentration. Compared to the ganglioside alone conditions, the addition of hIAPP further reduced cell viability for treatments with equimolar (10 μM) of any ganglioside and 10x (100 μM) GM3, but not for treatments with 10x (100 μM) GM1 or GD3. However, we cannot definitively conclude that the high ganglioside concentrations reduced the toxicity of hIAPP because the lipids were toxic themselves, and we could not distinguish between toxicity from hIAPP and toxicity from the gangliosides, particularly for GM1 and GD3.

## 4 DISCUSSION

Biophysical studies have extensively characterized hIAPP interactions with model lipid membranes containing various phospholipids (Caillon et al., 2013; Jayasinghe & Langen, 2007; Knight & Miranker, 2004; Lopes et al., 2007; Sasahara et al., 2010; Sciacca et al., 2012; Xing et al., 2017; Zhang, St Clair, et al., 2017). hIAPP binds bilayers composed of DOPG or POPG but not those consisting of DOPC or POPC, indicating a strong preference for negative charge density (Knight & Miranker, 2004; Lopes et al., 2007). Membranes composed of POPS or DOPS also strongly bind hIAPP, and simulations demonstrate that hIAPP possesses a greater affinity for mixed POPC/POPE membranes than for pure POPC membranes, confirming that this binding depends primarily on charge and not headgroup identity (Apostolidou et al., 2008; Caillon et al., 2013; Jayasinghe & Langen, 2007; Zhang, Ren, et al., 2017). One study used MD simulations to suggest that the charge on H18 contributes significantly to the electrostatics of lipid binding by hIAPP (Khemtemourian et al., 2022). Upon binding to anionic or zwitterionic phospholipid membranes and micelles, hIAPP adopts a partially *α*‐helical structure (Apostolidou et al., 2008; Brender, Hartman, et al., 2008; Brender, Lee, et al., 2008; Caillon et al., 2013; Jayasinghe & Langen, 2007; Knight et al., 2006; Lopes et al., 2007; Nanga et al., 2011; Patil et al., 2009). A low concentration of anionic phospholipids promotes hIAPP fibrillation, while a high concentration is inhibitory (Jayasinghe & Langen, 2007; Knight & Miranker, 2004). Similarly, vesicles with both zwitterionic and anionic lipids promote fibrillation with low anionic lipid content and inhibit with high anionic lipid content, suggesting that the effect of anionic lipids on hIAPP aggregation is independent of the presence of zwitterionic lipids (Jayasinghe & Langen, 2007; Knight & Miranker, 2004). The amount of anionic lipid required to inhibit hIAPP aggregation increases with the ionic strength of the solution, consistent with an electrostatic lipid‐peptide interaction (Jayasinghe & Langen, 2007).

**FIGURE 8:**
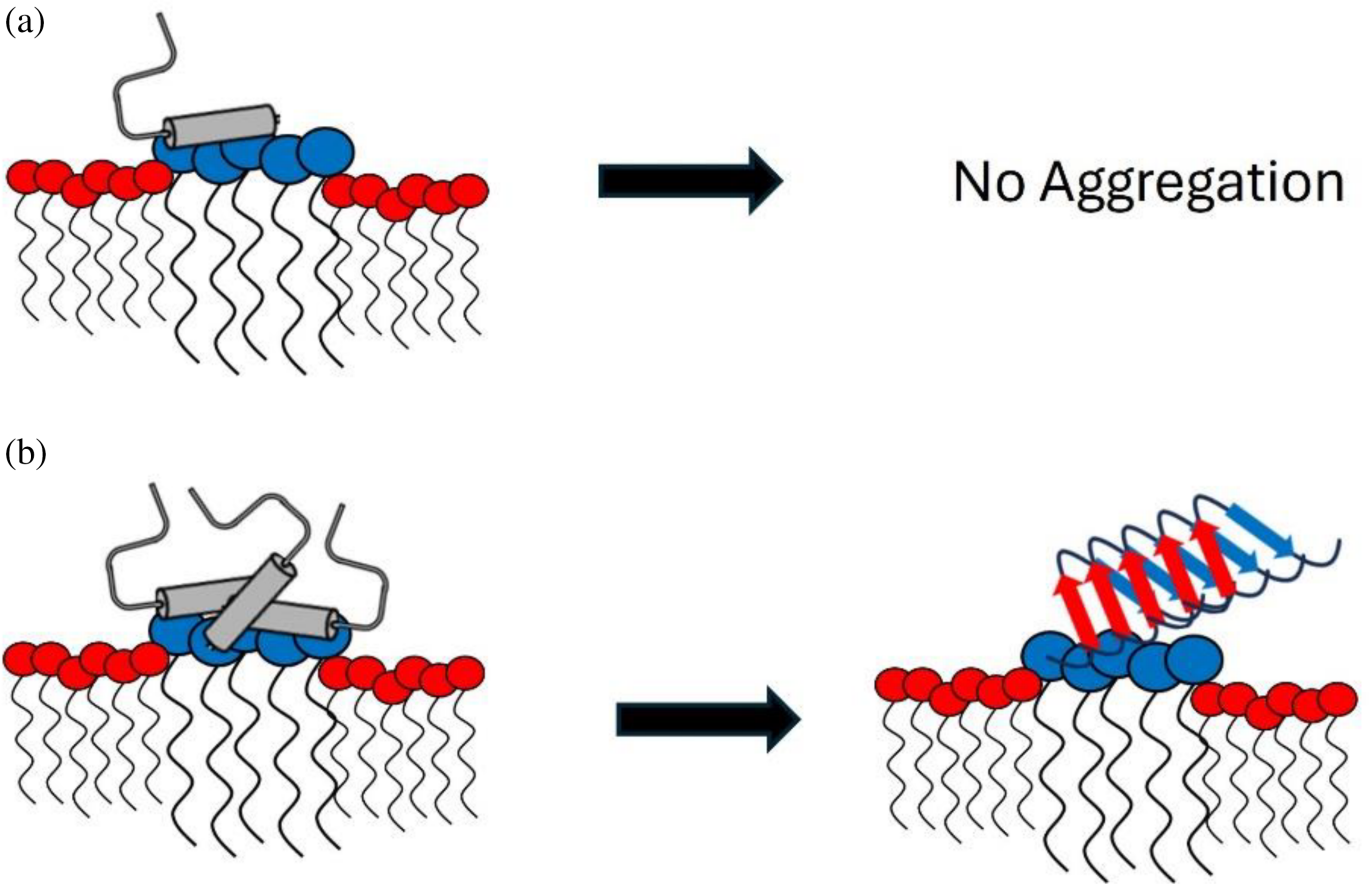
Schematic model for concentration‐dependent effects of gangliosides on human islet amyloid polypeptide (hIAPP) aggregation. (a) With a high ratio of ganglioside:peptide, there is sufficient area of ganglioside rafts (blue) in phospholipid (red) membranes for hIAPP monomers (gray) to bind diffusely, preventing aggregation. (b) In contrast, a low ganglioside:peptide ratio forces hIAPP monomers to bind in proximity to other monomer on scarce ganglioside rafts, facilitating self‐association for amyloid fibril formation.

We determined that the ganglioside lipids GM1, GM3, and GD3 exerted similar effects on hIAPP aggregation, dependent on the lipid: peptide ratio. As with the anionic phospholipids, the gangliosides promoted aggregation with low lipid:peptide but inhibited aggregation with high lipid:peptide. GD3, the most negatively charged ganglioside, most effectively promoted hIAPP aggregation, consistent with an electrostatic model of interaction. Similarly, the gangliosides, like other surfactants containing a negative charge, induced a *α*‐helix structure in hIAPP that converted to a *β*‐sheet over time with low lipid:peptide and was stable with high lipid:peptide (Jayasinghe & Langen, 2007; Nanga et al., 2011). These opposing effects could be explained by considering the available lipid surface area for binding by hIAPP (Figure 8). At high lipid concentrations, more liposomes or micelles provide a greater available lipid surface area for hIAPP binding. As a result, hIAPP can bind more diffusely and is thus less likely to self‐associate. Reducing the lipid concentration reduces the available binding area for hIAPP, thereby increasing the spatial proximity of lipid‐bound hIAPP monomers and facilitating their interaction. GM3 required the highest lipid:peptide ratio to inhibit hIAPP aggregation, GD3 the next highest, and GM1 the least. This is in order of the number of headgroup carbohydrate moieties, supporting the idea that lipid surface area facilitates their inhibition of hIAPP aggregation. The lipid surface area likely stabilizes hIAPP by promoting hydrogen bonding with ganglioside headgroups instead of with other hIAPP molecules, as was shown for A*β* (Fatafta et al., 2021).

Using TEM, we also observed that the gangliosides altered the morphology of hIAPP fibrils. In the presence of each ganglioside, an amorphous density formed along the hIAPP fibril length. Fibrils with a similar morphology have been observed following hIAPP aggregation on phospholipid membranes, and the amorphous density has been attributed to lipids coating the fibril surface, though this lipid wrapping has not always been observed under such conditions (Domanov & Kinnunen, 2008; Knight & Miranker, 2004; Sparr et al., 2004). hIAPP also modified the aggregation of the lipids, preventing the formation of worm‐like tubules of GM1 and GM3 at high lipid concentrations. Previous work has shown that hIAPP senses membrane curvature and remodels membranes (Kegulian et al., 2015; Smith et al., 2009). For instance, hIAPP transformed large POPS vesicles into smaller liposomes, like what we reported (Kegulian et al., 2015). These observations may support mechanisms by which hIAPP can disrupt ganglioside‐containing plasma membranes, leading to cell death. Lipid‐wrapped fibrils suggest that hIAPP aggregates could sequester lipids from the membrane, as in the proposed detergent‐like mechanism of membrane disruption by hIAPP (Sciacca et al., 2021). Alternatively, hIAPP might generate local regions of high curvature in the cell membrane, causing stress that ultimately results in perforation of the cell (Smith et al., 2009).

In summary, we studied the effects of the gangliosides GM1, GM3, and GD3 on hIAPP aggregation. For each ganglioside, equimolar or lower concentrations relative to hIAPP promoted aggregation, and GD3 was more effective than GM1 or GM3. On the other hand, higher ganglioside concentrations, relative to hIAPP, inhibited hIAPP aggregation. GM1 and GD3 were more efficient inhibitors than GM3, likely due to the larger headgroups providing greater surface area for hIAPP binding. We also described changes in hIAPP and ganglioside aggregate morphologies that potentially support curvature‐strain‐induced and detergent‐like mechanisms of hIAPP‐induced membrane penetration. However, more work is needed to clarify the molecular underpinnings of membrane disruption by hIAPP. Thus, further research should expand the scope of this report to include physiologically relevant model membranes with zwitterionic phospholipids, cholesterol, and ganglioside rafts. More work is also needed to elucidate the nature and structure of hIAPP aggregates that form in the presence of ganglioside‐enriched membranes and how these species correlate with the cytotoxicity associated with T2D. Lastly, the toxic effects of hIAPP on cells and the effects of ganglioside‐enriched membranes will require substantial work and additional experiments to fully elucidate. Some research indicates that hIAPP aggregation causes toxicity to cells by promoting apoptosis, so characterizations of apoptosis, such as with immunostaining‐based assays of caspase activation, might provide a fuller picture of amyloid‐induced cytotoxicity than the MTT assay (Bram et al., 2014; Jurgens et al., 2011; Lorenzo et al., 1994). Such studies could provide a fuller picture of the toxic nature of hIAPP aggregates and inspire the development of more effective therapeutics against T2D.

## AUTHOR CONTRIBUTIONS

**Samuel D. McCalpin:** Conceptualization; methodology; investigation; formal analysis; writing – original draft; writing – review and editing; data curation. **Lina Mechakra:** Investigation. **Magdalena I. Ivanova:** Conceptualization; methodology; investigation; supervision; writing – review and editing; project administration; resources. **Ayyalusamy Ramamoorthy:** Conceptualization; methodology; investigation; writing – review and editing; project administration; funding acquisition; supervision; resources.

## Supporting information


**Data S1.** Supporting information.

## References

[pro5119-bib-0001] Abedini A , Cao P , Plesner A , Zhang J , He M , Derk J , et al. RAGE binds Preamyloid IAPP intermediates and mediates pancreatic β cell proteotoxicity. J Clin Invest. 2018;128(2):682–698. 10.1172/JCI85210 29337308 PMC5785261

[pro5119-bib-0002] Abedini A , Plesner A , Cao P , Ridgway Z , Zhang J , Tu L‐H , et al. Time‐resolved studies define the nature of toxic IAPP intermediates, providing insight for anti‐amyloidosis therapeutics. eLife. 2016;5:e12977. 10.7554/eLife.12977 27213520 PMC4940161

[pro5119-bib-0003] Apostolidou M , Jayasinghe SA , Langen R . Structure of α‐helical membrane‐bound human islet amyloid polypeptide and its implications for membrane‐mediated misfolding. J Biol Chem. 2008;283(25):17205–17210. 10.1074/jbc.M801383200 18442979 PMC2427348

[pro5119-bib-0004] Baba M , Nakajo S , Tu PH , Tomita T , Nakaya K , Lee VM , et al. Aggregation of alpha‐synuclein in Lewy bodies of sporadic Parkinson's disease and dementia with Lewy bodies. Am J Pathol. 1998;152(4):879–884. 9546347 PMC1858234

[pro5119-bib-0005] Betsholtz C , Christmansson L , Engström U , Rorsman F , Svensson V , Johnson KH , et al. Sequence divergence in a specific region of islet amyloid polypeptide (IAPP) explains differences in islet amyloid formation between species. FEBS Lett. 1989;251(1–2):261–264. 10.1016/0014-5793(89)81467-X 2666169

[pro5119-bib-0006] Betsholtz C , Svensson V , Rorsman F , Engström U , Westermark GT , Wilander E , et al. Islet amyloid polypeptide (IAPP): cDNA cloning and identification of an amyloidogenic region associated with the species‐specific occurrence of age‐related diabetes mellitus. Exp Cell Res. 1989;183(2):484–493. 10.1016/0014-4827(89)90407-2 2670595

[pro5119-bib-0007] Bram Y , Frydman‐Marom A , Yanai I , Gilead S , Shaltiel‐Karyo R , Amdursky N , et al. Apoptosis induced by islet amyloid polypeptide soluble oligomers is neutralized by diabetes‐associated specific antibodies. Sci Rep. 2014;4(1):4267. 10.1038/srep04267 24589570 PMC3940978

[pro5119-bib-0008] Brender JR , Hartman K , Reid KR , Kennedy RT , Ramamoorthy A . A single mutation in the nonamyloidogenic region of islet amyloid polypeptide greatly reduces toxicity. Biochemistry. 2008;47(48):12680–12688. 10.1021/bi801427c 18989933 PMC2645932

[pro5119-bib-0009] Brender JR , Lee EL , Cavitt MA , Gafni A , Steel DG , Ramamoorthy A . Amyloid fiber formation and membrane disruption are separate processes localized in two distinct regions of IAPP, the type‐2‐diabetes‐related peptide. J Am Chem Soc. 2008;130(20):6424–6429. 10.1021/ja710484d 18444645 PMC4163023

[pro5119-bib-0010] Caillon L , Lequin O , Khemtémourian L . Evaluation of membrane models and their composition for islet amyloid polypeptide‐membrane aggregation. Biochim Biophys Acta BBA—Biomembr. 2013;1828(9):2091–2098. 10.1016/j.bbamem.2013.05.014 23707907

[pro5119-bib-0011] Chakravorty A , McCalpin SD , Sahoo BR , Ramamoorthy A , Brooks CL . Free gangliosides can Alter amyloid‐β aggregation. J Phys Chem Lett. 2022;13:9303–9308. 10.1021/acs.jpclett.2c02362 36174129 PMC9700483

[pro5119-bib-0012] Christensen M , Schiøtt B . Revealing a dual role of ganglioside lipids in the aggregation of membrane‐associated islet amyloid polypeptide. J Membr Biol. 2019;252(4):343–356. 10.1007/s00232-019-00074-5 31222470

[pro5119-bib-0013] Corti M , Degiorgio V , Ghidoni R , Sonnino S . Micellar properties of gangliosides. In: Mittal KL , Fendler EJ , editors. Solution behavior of surfactants: theoretical and applied aspects. Volume 1. Boston, MA: Springer US; 1982. p. 573–594. 10.1007/978-1-4613-3491-0_30

[pro5119-bib-0014] Corti M , Degiorgio V , Ghidoni R , Sonnino S , Tettamanti G . Laser‐light scattering investigation of the micellar properties of gangliosides. Chem Phys Lipids. 1980;26(3):225–238. 10.1016/0009-3084(80)90053-5 7371117

[pro5119-bib-0015] Di Paolo G , De Camilli P . Phosphoinositides in cell regulation and membrane dynamics. Nature. 2006;443(7112):651–657. 10.1038/nature05185 17035995

[pro5119-bib-0016] Dodge JT , Phillips GB . Composition of phospholipids and of phospholipid fatty acids and aldehydes in human red cells. J Lipid Res. 1967;8(6):667–675. 6057495

[pro5119-bib-0017] Doktorova M , Symons JL , Zhang X , Wang H‐Y , Schlegel J , Lorent JH , et al. Cell membranes sustain phospholipid imbalance via cholesterol asymmetry. bioRxiv. 2023;31:551157. 10.1101/2023.07.30.551157 PMC12085300 40179882

[pro5119-bib-0018] Domanov YA , Kinnunen PKJ . Islet amyloid polypeptide forms rigid lipid–protein amyloid fibrils on supported phospholipid bilayers. J Mol Biol. 2008;376(1):42–54. 10.1016/j.jmb.2007.11.077 18155730

[pro5119-bib-0019] Dotta F , Colman PG , Lombardi D , Scharp DW , Andreani D , Pontieri GM , et al. Ganglioside expression in human pancreatic islets. Diabetes. 1989;38(11):1478–1483. 10.2337/diab.38.11.1478 2695376

[pro5119-bib-0020] Dotta F , Tiberti C , Previti M , Anastasi E , Andreani D , Lenti L , et al. Rat pancreatic ganglioside expression: differences between a model of autoimmune islet B cell destruction and a Normal strain. Clin Immunol Immunopathol. 1993;66(2):143–149. 10.1006/clin.1993.1018 8453786

[pro5119-bib-0021] Fatafta H , Khaled M , Owen MC , Sayyed‐Ahmad A , Strodel B . Amyloid‐β peptide dimers undergo a random coil to β‐sheet transition in the aqueous phase but not at the neuronal membrane. Proc Natl Acad Sci. 2021;118(39):e2106210118. 10.1073/pnas.2106210118 34544868 PMC8488611

[pro5119-bib-0022] Feizi T . Demonstration by monoclonal antibodies that carbohydrate structures of glycoproteins and glycolipids are onco‐developmental antigens. Nature. 1985;314(6006):53–57. 10.1038/314053a0 2579340

[pro5119-bib-0023] Formisano S , Johnson ML , Lee G , Aloj SM , Edelhoch H . Critical micelle concentrations of gangliosides. Biochemistry. 1979;18(6):1119–1124. 10.1021/bi00573a028 570850

[pro5119-bib-0024] Haataja L , Gurlo T , Huang CJ , Butler PC . Islet amyloid in type 2 diabetes, and the toxic oligomer hypothesis. Endocr Rev. 2008;29(3):303–316. 10.1210/er.2007-0037 18314421 PMC2528855

[pro5119-bib-0025] Hickey AJR , Bradley JWI , Skea GL , Middleditch MJ , Buchanan CM , Phillips ARJ , et al. Proteins associated with immunopurified granules from a model pancreatic islet β‐cell system: proteomic snapshot of an endocrine secretory granule. J Proteome Res. 2009;8(1):178–186. 10.1021/pr800675k 19055480

[pro5119-bib-0026] Hoenig M . The cat as a model for human obesity and diabetes. J Diabetes Sci Technol. 2012;6(3):525–533. 10.1177/193229681200600306 22768882 PMC3440058

[pro5119-bib-0027] Howard CF Jr . Insular amyloidosis and diabetes mellitus in Macaca Nigra. Diabetes. 1978;27(4):357–364. 10.2337/diab.27.4.357 416984

[pro5119-bib-0028] Ingólfsson HI , Melo MN , van Eerden FJ , Arnarez C , Lopez CA , Wassenaar TA , et al. Lipid organization of the plasma membrane. J Am Chem Soc. 2014;136(41):14554–14559. 10.1021/ja507832e 25229711

[pro5119-bib-0029] Janson J , Ashley RH , Harrison D , McIntyre S , Butler PC . The mechanism of islet amyloid polypeptide toxicity is membrane disruption by intermediate‐sized toxic amyloid particles. Diabetes. 1999;48(3):491–498. 10.2337/diabetes.48.3.491 10078548

[pro5119-bib-0030] Janson J , Soeller WC , Roche PC , Nelson RT , Torchia AJ , Kreutter DK , et al. Spontaneous diabetes mellitus in transgenic mice expressing human islet amyloid polypeptide. Proc Natl Acad Sci. 1996;93(14):7283–7288. 10.1073/pnas.93.14.7283 8692984 PMC38975

[pro5119-bib-0031] Jayasinghe SA , Langen R . Membrane interaction of islet amyloid polypeptide. Biochim Biophys Acta BBA—Biomembr. 2007;1768(8):2002–2009. 10.1016/j.bbamem.2007.01.022 17349968

[pro5119-bib-0032] Jiang W , Chen H , Yang L , Pan X . moreThanANOVA: a user‐friendly shiny/R application for exploring and comparing data with interactive visualization. PLoS ONE. 2022;17(7):e0271185. 10.1371/journal.pone.0271185 35802729 PMC9269871

[pro5119-bib-0033] Jurgens CA , Toukatly MN , Fligner CL , Udayasankar J , Subramanian SL , Zraika S , et al. β‐Cell loss and β‐cell apoptosis in human type 2 diabetes are related to islet amyloid deposition. Am J Pathol. 2011;178(6):2632–2640. 10.1016/j.ajpath.2011.02.036 21641386 PMC3123989

[pro5119-bib-0034] Kapurniotu A . Amyloidogenicity and cytotoxicity of islet amyloid polypeptide. Pept Sci. 2001;60(6):438–459. 10.1002/1097-0282(2001)60:6<438::AID-BIP10182>3.0.CO;2-A 12209476

[pro5119-bib-0035] Kegulian NC , Sankhagowit S , Apostolidou M , Jayasinghe SA , Malmstadt N , Butler PC , et al. Membrane curvature‐sensing and curvature‐inducing activity of islet amyloid polypeptide and its implications for membrane disruption. J Biol Chem. 2015;290(43):25782–25793. 10.1074/jbc.M115.659797 26283787 PMC4646232

[pro5119-bib-0036] Khemtemourian L , Fatafta H , Davion B , Lecomte S , Castano S , Strodel B . Structural dissection of the first events following membrane binding of the islet amyloid polypeptide. Front Mol Biosci. 2022;9. 10.3389/fmolb.2022.849979 PMC8965455 35372496

[pro5119-bib-0037] Knight JD , Hebda JA , Miranker AD . Conserved and cooperative assembly of membrane‐bound α‐helical states of islet amyloid polypeptide. Biochemistry. 2006;45(31):9496–9508. 10.1021/bi060579z 16878984

[pro5119-bib-0038] Knight JD , Miranker AD . Phospholipid catalysis of diabetic amyloid assembly. J Mol Biol. 2004;341(5):1175–1187. 10.1016/j.jmb.2004.06.086 15321714

[pro5119-bib-0039] Kolter T . Ganglioside biochemistry. ISRN Biochem. 2012;2012:506160. 10.5402/2012/506160 25969757 PMC4393008

[pro5119-bib-0040] Ledeen RW , Wu G . Chapter fifteen—gangliosides, α‐synuclein, and Parkinson's disease. In: Schnaar RL , Lopez PHH , editors. Progress in molecular biology and translational science. Volume 156. Gangliosides in Health and Disease; Academic Press; 2018. p. 435–454. 10.1016/bs.pmbts.2017.12.009 29747823

[pro5119-bib-0041] Lopes DHJ , Meister A , Gohlke A , Hauser A , Blume A , Winter R . Mechanism of islet amyloid polypeptide fibrillation at lipid interfaces studied by infrared reflection absorption spectroscopy. Biophys J. 2007;93(9):3132–3141. 10.1529/biophysj.107.110635 17660321 PMC2025658

[pro5119-bib-0042] Lorenzo A , Razzaboni B , Weir GC , Yankner BA . Pancreatic islet cell toxicity of amylin associated with type‐2 diabetes mellitus. Nature. 1994;368(6473):756–760. 10.1038/368756a0 8152488

[pro5119-bib-0043] Marzban L , Park K , Verchere CB . Islet amyloid polypeptide and type 2 diabetes. Exp Gerontol. 2003;38(4):347–351. 10.1016/S0531-5565(03)00004-4 12670620

[pro5119-bib-0044] Matsuzaki K . How do membranes initiate Alzheimer's disease? Formation of toxic amyloid fibrils by the amyloid β‐protein on ganglioside clusters. Acc Chem Res. 2014;47(8):2397–2404. 10.1021/ar500127z 25029558

[pro5119-bib-0045] Matveyenko AV , Butler PC . Islet amyloid polypeptide (IAPP) transgenic rodents as models for type 2 diabetes. ILAR J. 2006;47(3):225–233. 10.1093/ilar.47.3.225 16804197

[pro5119-bib-0046] Meisl G , Kirkegaard JB , Arosio P , Michaels TCT , Vendruscolo M , Dobson CM , et al. Molecular mechanisms of protein aggregation from global fitting of kinetic models. Nat Protoc. 2016;11(2):252–272. 10.1038/nprot.2016.010 26741409

[pro5119-bib-0047] Micsonai A , Moussong É , Wien F , Boros E , Vadászi H , Murvai N , et al. BeStSel: webserver for secondary structure and fold prediction for protein CD spectroscopy. Nucleic Acids Res. 2022;50(W1):W90–W98. 10.1093/nar/gkac345 35544232 PMC9252784

[pro5119-bib-0048] Milardi D , Gazit E , Radford SE , Xu Y , Gallardo RU , Caflisch A , et al. Proteostasis of islet amyloid polypeptide: a molecular perspective of risk factors and protective strategies for type II diabetes. Chem Rev. 2021;121(3):1845–1893. 10.1021/acs.chemrev.0c00981 33427465 PMC10317076

[pro5119-bib-0049] Mosselman S , Höppener J , Lips CJM , Jansz HS . The complete islet amyloid polypeptide precursor is encoded by two exons. FEBS Lett. 1989;247(1):154–158. 10.1016/0014-5793(89)81260-8 2651160

[pro5119-bib-0050] Nanga RPR , Brender JR , Vivekanandan S , Ramamoorthy A . Structure and membrane orientation of IAPP in its natively amidated form at physiological pH in a membrane environment. Biochim Biophys Acta BBA—Biomembr. 2011;1808(10):2337–2342. 10.1016/j.bbamem.2011.06.012 PMC3156962 21723249

[pro5119-bib-0051] Nauck MA , Wefers J , Meier JJ . Treatment of type 2 diabetes: challenges, hopes, and anticipated successes. Lancet Diabetes Endocrinol. 2021;9(8):525–544. 10.1016/S2213-8587(21)00113-3 34181914

[pro5119-bib-0052] Nishi M , Chan SJ , Nagamatsu S , Bell GI , Steiner DF . Conservation of the sequence of islet amyloid polypeptide in five mammals is consistent with its putative role as an islet hormone. Proc Natl Acad Sci. 1989;86(15):5738–5742. 10.1073/pnas.86.15.5738 2668946 PMC297705

[pro5119-bib-0053] Ong KL , Stafford LK , McLaughlin SA , Boyko EJ , Vollset SE , Smith AE , et al. Global, regional, and National Burden of diabetes from 1990 to 2021, with projections of prevalence to 2050: a systematic analysis for the global burden of disease study 2021. Lancet. 2023;402(10397):203–234. 10.1016/S0140-6736(23)01301-6 37356446 PMC10364581

[pro5119-bib-0054] Op den Kamp JAF . Lipid asymmetry in membranes. Annu Rev Biochem. 1979;48(1):47–71. 10.1146/annurev.bi.48.070179.000403 382989

[pro5119-bib-0055] Oshima H , Soma G‐I , Mizuno D . Gangliosides can activate human alternative complement pathway. Int Immunol. 1993;5(10):1349–1351. 10.1093/intimm/5.10.1349 8268140

[pro5119-bib-0056] Palato LM , Pilcher S , Oakes A , Lamba A , Torres J , Ledesma Monjaraz LI , et al. Amyloidogenicity of naturally occurring full‐length animal IAPP variants. J Pept Sci. 2019;25(8):e3199. 10.1002/psc.3199 31231935 PMC6639132

[pro5119-bib-0057] Patil SM , Xu S , Sheftic SR , Alexandrescu AT . Dynamic α‐helix structure of micelle‐bound human amylin. J Biol Chem. 2009;284(18):11982–11991. 10.1074/jbc.M809085200 19244249 PMC2673267

[pro5119-bib-0058] Raleigh D , Zhang X , Hastoy B , Clark A . The β‐cell assassin: IAPP cytotoxicity. J Mol Endocrinol. 2017;59(3):R121–R140. 10.1530/JME-17-0105 28811318

[pro5119-bib-0059] Rauvala H . Monomer‐micelle transition of the ganglioside GM1 and the hydrolysis by clostridium perfringens neuraminidase. Eur J Biochem. 1979;97(2):555–564. 10.1111/j.1432-1033.1979.tb13144.x 467431

[pro5119-bib-0060] Saha J , Ford BJ , Wang X , Boyd S , Morgan SE , Rangachari V . Sugar distributions on gangliosides guide the formation and stability of amyloid‐β oligomers. Biophys Chem. 2023;300:107073. 10.1016/j.bpc.2023.107073 37413816 PMC10529042

[pro5119-bib-0061] Saito M , Sugiyama K . A distinct ganglioside composition of rat pancreatic islets. Arch Biochem Biophys. 2000;376(2):371–376. 10.1006/abbi.2000.1729 10775425

[pro5119-bib-0062] Sanke T , Bell GI , Sample C , Rubenstein AH , Steiner DF . An islet amyloid peptide is derived from an 89‐amino acid precursor by proteolytic processing. J Biol Chem. 1988;263(33):17243–17246. 10.1016/S0021-9258(19)77825-9 3053705

[pro5119-bib-0063] Sasahara K , Hall D , Hamada D . Effect of lipid type on the binding of lipid vesicles to islet amyloid polypeptide amyloid fibrils. Biochemistry. 2010;49(14):3040–3048. 10.1021/bi9019252 20210361

[pro5119-bib-0064] Sciacca MFM , Brender JR , Lee D‐K , Ramamoorthy A . Phosphatidylethanolamine enhances amyloid fiber‐dependent membrane fragmentation. Biochemistry. 2012;51(39):7676–7684. 10.1021/bi3009888 22970795 PMC3464957

[pro5119-bib-0065] Sciacca MFM , La Rosa C , Milardi D . Amyloid‐mediated mechanisms of membrane disruption. Biophysica. 2021;1(2):137–156. 10.3390/biophysica1020011

[pro5119-bib-0066] Sepehri A , Nepal B , Lazaridis T . Distinct modes of action of IAPP oligomers on membranes. J Chem Inf Model. 2021;61(9):4645–4655. 10.1021/acs.jcim.1c00767 34499498

[pro5119-bib-0067] Serravalle S , Pisano M , Sciacca MFM , Salamone N , Sicali L , Mazzara G , et al. Critical micellar concentration determination of pure phospholipids and lipid‐raft and their mixtures with cholesterol. Proteins Struct. Funct. Bioinforma. 2024. 10.1002/prot.26669 PMC12260274 38234101

[pro5119-bib-0068] Smith PES , Brender JR , Ramamoorthy A . Induction of negative curvature as a mechanism of cell toxicity by amyloidogenic peptides: the case of islet amyloid polypeptide. J Am Chem Soc. 2009;131(12):4470–4478. 10.1021/ja809002a 19278224 PMC2665920

[pro5119-bib-0069] Sparr E , Engel MFM , Sakharov DV , Sprong M , Jacobs J , de Kruijff B , et al. Islet amyloid polypeptide‐induced membrane leakage involves uptake of lipids by forming amyloid fibers. FEBS Lett. 2004;577(1–2):117–120. 10.1016/j.febslet.2004.09.075 15527771

[pro5119-bib-0070] Tempra C , Scollo F , Pannuzzo M , Lolicato F , La Rosa C . A unifying framework for amyloid‐mediated membrane damage: the lipid‐chaperone hypothesis. Biochim Biophys Acta BBA—Proteins Proteomics. 2022;1870(4):140767. 10.1016/j.bbapap.2022.140767 35144022

[pro5119-bib-0071] van Meer G , Voelker DR , Feigenson GW . Membrane lipids: where they are and how they behave. Nat Rev Mol Cell Biol. 2008;9(2):112–124. 10.1038/nrm2330 18216768 PMC2642958

[pro5119-bib-0072] Verkleij AJ , Zwaal RFA , Roelofsen B , Comfurius P , Kastelijn D , van Deenen LLM . The asymmetric distribution of phospholipids in the human red cell membrane. A combined study using phospholipases and freeze‐etch electron microscopy. Biochim Biophys Acta BBA—Biomembr. 1973;323(2):178–193. 10.1016/0005-2736(73)90143-0 4356540

[pro5119-bib-0073] Virtanen JA , Cheng KH , Somerharju P . Phospholipid composition of the mammalian red cell membrane can be rationalized by a superlattice model. Proc Natl Acad Sci. 1998;95(9):4964–4969. 10.1073/pnas.95.9.4964 9560211 PMC20196

[pro5119-bib-0074] Wakabayashi M , Matsuzaki K . Ganglioside‐induced amyloid formation by human islet amyloid polypeptide in lipid rafts. FEBS Lett. 2009;583(17):2854–2858. 10.1016/j.febslet.2009.07.044 19647738

[pro5119-bib-0075] Xing Y , Pilkington EH , Wang M , Nowell CJ , Kakinen A , Sun Y , et al. Lysophosphatidylcholine modulates the aggregation of human islet amyloid polypeptide. Phys Chem Chem Phys. 2017;19(45):30627–30635. 10.1039/C7CP06670H 29115353 PMC5901975

[pro5119-bib-0076] Yohe HC , Rosenberg A . Interaction of triiodide anion with gangliosides in aqueous iodine. Chem Phys Lipids. 1972;9(4):279–294. 10.1016/0009-3084(72)90015-1 4646538

[pro5119-bib-0077] Zhang M , Ren B , Liu Y , Liang G , Sun Y , Xu L , et al. Membrane interactions of hIAPP monomer and oligomer with lipid membranes by molecular dynamics simulations. ACS Chem Nerosci. 2017;8(8):1789–1800. 10.1021/acschemneuro.7b00160 28585804

[pro5119-bib-0078] Zhang X , St Clair JR , London E , Raleigh DP . Islet amyloid polypeptide membrane interactions: effects of membrane composition. Biochemistry. 2017;56(2):376–390. 10.1021/acs.biochem.6b01016 28054763 PMC5541234

[pro5119-bib-0079] Zwaal RFA , Roelofsen B , Comfurius P , van Deenen LLM . Organization of phospholipids in human red cell membranes as detected by the action of various purified phospholipases. Biochim Biophys Acta BBA—Biomembr. 1975;406(1):83–96. 10.1016/0005-2736(75)90044-9 169915

